# Different definitions of CpG island methylator phenotype and outcomes of colorectal cancer: a systematic review

**DOI:** 10.1186/s13148-016-0191-8

**Published:** 2016-03-02

**Authors:** Min Jia, Xu Gao, Yan Zhang, Michael Hoffmeister, Hermann Brenner

**Affiliations:** Division of Clinical Epidemiology and Aging Research, German Cancer Research Center (DKFZ), Heidelberg, Germany; German Cancer Consortium (DKTK), Heidelberg, Germany

**Keywords:** Colorectal cancer, CpG island methylator phenotype, Prognosis, Chemotherapy

## Abstract

Contradictory results were reported for the prognostic role of CpG island methylator phenotype (CIMP) among colorectal cancer (CRC) patients. Differences in the definitions of CIMP were the most common explanation for these discrepancies. The aim of this systematic review was to give an overview of the published studies on CRC prognosis according to the different definitions of CIMP. A systematic literature search was performed in MEDLINE and ISI Web of Science for articles published until 3 April 2015. Data extraction included information about the study population, the definition of CIMP, and investigated outcomes. Thirty-six studies were included in this systematic review. Among them, 30 studies reported the association of CIMP and CRC prognosis and 11 studies reported the association of CIMP with survival after CRC therapy. Overall, 16 different definitions of CIMP were identified. The majority of studies reported a poorer prognosis for patients with CIMP-positive (CIMP+)/CIMP-high (CIMP-H) CRC than with CIMP-negative (CIMP−)/CIMP-low (CIMP-L) CRC. Inconsistent results or varying effect strengths could not be explained by different CIMP definitions used. No consistent variation in response to specific therapies according to CIMP status was found. Comparative analyses of different CIMP panels in the same large study populations are needed to further clarify the role of CIMP definitions and to find out how methylation information can best be used to predict CRC prognosis and response to specific CRC therapies.

**Electronic supplementary material:**

The online version of this article (doi:10.1186/s13148-016-0191-8) contains supplementary material, which is available to authorized users.

## Background

With estimated numbers of approximately 1.4 million new cases per year, colorectal cancer (CRC) is the third most commonly diagnosed cancer in males and the second most common in females globally [1]. The 5-year survival rate is less than 65 % [2].

CpG island methylator phenotype (CIMP) which was originally introduced by Toyata and colleagues in 1999 [3] is characterized by simultaneous hypermethylation of numerous CpG islands surrounding the promoter regions of several genes [4]. Methylation of CpG islands in the promoter of tumor-suppressor genes could physically inhibit binding of transcription factors [5]. By transcriptional silencing of these genes, CIMP is believed to contribute to the onset and progression of CRC [6, 7]. However, definitions of CIMP varied widely between studies with respect to methylation loci considered and laboratory methods used to determine methylation.

Previous studies showed that CIMP is associated with altered molecular and clinical characteristics [8, 9]. However, the prognostic effect of CIMP in CRC was not consistent in previous studies. Recently, Juo and colleagues [10] conducted a meta-analysis supporting the hypothesis that CIMP positivity predicts poorer survival among patients with CRC. However, this meta-analysis did not take the differences in the definitions of CIMP into consideration. The aim of this systematic review was to give an overview of the published studies on CRC outcomes according to the definitions of CIMP and to provide a deeper understanding of the importance of CIMP definition in the evaluation of CRC prognosis.

## Methods

### Search strategy

Our systematic literature search was performed in accordance with PRISMA recommendations [11]. PubMed and ISI Web of Knowledge databases were used for the search of relevant articles from inception to 3 April 2015, using neither filters nor language restrictions. The combination of keywords used was [colorectal (or) colon (or) rectum] (and) [cancer (or) neoplasm (or) carcinoma (or) adenoma (or) malignancy] (and) [methylation] (and) [prognosis (or) prognostic (or) survival (or) follow up (or) mortality (or) long term] (and) [CIMP (or) CpG island methylator phenotype]. A search for additional relevant studies was carried out in the reference lists of the identified studies.

### Eligibility criteria

After deleting duplicate articles, each title and abstract was checked for relevant content. Only studies published in English language that were conducted in human beings, measuring methylation in a biological sample, were included. Studies not relevant to the topic, such as studies only testing global DNA methylation level in human blood or cell lines, were excluded. Since detailed information was needed for further review, studies with only conference abstract or not reported in original articles were also excluded. As this review focused on CRC outcomes according to different CIMP definitions, studies not reporting the specific methylation markers used for the definition of CIMP or the criteria for the classification of CIMP categories and studies that did not have survival data of CRC patients were excluded after full-text review.

### Data extraction

Data were extracted from the eligible studies by two investigators (MJ and XG) independently into data extraction tables. Any disagreement was resolved by consensus after further review of the original text. The following data items were extracted: study population information (authors, year of publication, country, patient population, number of subjects, age, sex, and follow-up time), information about the definition of CIMP (methylation loci, classification of CIMP, prevalence of CIMP, and laboratory method for methylation analysis), and outcome data (*p* value and hazard ratio (HR) and 95 % confidence intervals (95 % CI)) including associations with overall survival (OS), disease-specific survival (DSS), disease-free survival (DFS), and recurrence-free survival (RFS). Given the importance of potential confounding factors, we extracted data from the most comprehensively adjusted model only when results from different models were reported. If no adjusted results were available, we included the unadjusted results.

## Results

### Literature search result

**Fig. 1:**
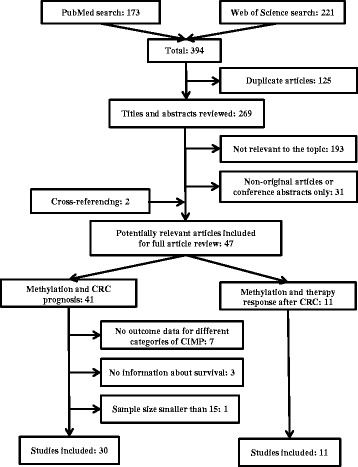
Flow diagram of the literature search process and the studies included in this systematic review

The literature search found 394 articles in total (Fig. 1). After removal of 125 duplicates, 269 titles and abstracts were screened for potentially relevant articles. Following exclusion of 193 non-relevant articles and 31 non-original articles, 45 studies were left for full-text review, and two additional studies related to the topic were found by cross-referencing. These 47 studies included studies investigating the association between CIMP and prognosis of CRC (*n* = 41) and studies investigating survival after specific CRC therapies according to CIMP status (*n* = 11). Among the 41 studies on CRC prognosis, 11 studies were excluded after full-text assessment for the following reasons: studies with no comparison of different CIMP categories with respect to prognosis (*n* = 7); studies without information about survival (*n* = 3); and studies with too small sample size to carry out meaningful survival analyses (*n* = 1). Finally, we included 30 studies on the association of CIMP with CRC prognosis and 11 studies on the association of CIMP with survival according to CRC therapy.

### Study population characteristics

An overview of the 30 included studies on CIMP and prognosis of CRC [4, 8, 12–40] is shown in Additional file 1: Table S1. Most studies investigated colon and rectal cancer patients (26/30), whereas others only focused on colon cancer patients (4/30) or rectal cancer patients (1/30). Some of the studies were restricted to specific stages of CRC (*n* = 5) or specific molecular subtypes of CRC (3/30). OS was assessed in most of the studies, while others assessed DFS, DSS, and RFS as the primary end point. The 11 studies on survival after specific CRC therapies according to CIMP status [4, 25, 34–36, 40–45] were mainly including stage II or stage III CRC patients (Additional file 2: Table S2). Six of these 11 studies focused on patients with surgery alone and patients with surgery and adjuvant chemotherapy [4, 25, 34, 36, 40, 41]. One study [35] investigated two patient groups with different chemotherapy regimens after surgery. The remaining four studies enrolled patients treated with chemotherapy only or with chemotherapy after surgery [42–45].

### CIMP definition

Gene panels used for the definition of CIMP varied between the studies (Tables 1 and 2). CIMP was either classified in two groups (CIMP positive (CIMP+) and CIMP negative (CIMP−)) or in three groups (CIMP-high (CIMP-H), CIMP-low (CIMP-L), and CIMP-negative (CIMP-N)). In the 16 different marker panels used for the definition of CIMP, the number of methylation loci included ranged between 5 and 15. Additionally, definitions 1 and 2 and definitions 3 and 4 used different cutoff values for CIMP-H or CIMP+. Prevalence of CIMP+ or CIMP-H varied between different CIMP definitions and the patient samples investigated (6.4–48.5 %). The difference of prevalence of CIMP+ or CIMP-H among studies using the same CIMP definition might be due to different patient races, different laboratory methods, and different subgroups of CRC patients. Regarding the laboratory method used to analyze the methylation of genes, methylation-specific PCR (MSP, *n* = 15) [46] and MethyLight (*n* = 16) [47] were the most frequently used. Other methods such as pyrosequencing (*n* = 2), MassARRAY (*n* = 1) [22, 48], MS-HRM (methylation-sensitive high-resolution melting, *n* = 1) [49], and COBRA (combined bisulfite restriction analysis, *n* = 1) [43] were used by few studies only (Additional file 3: Table S3 and Additional file 4: Table S4). Where reported, DNA was extracted from formalin-fixed and paraffin-embedded (FFPE) tissue in the majority of studies (Additional file 3: Table S3 and Additional file 4: Table S4).

**Table 1:** CIMP definitions and prevalence in studies on colorectal cancer prognosis

Definition	First author (year)	Common CIMP genes											Other CIMP genes	CIMP category			CIMP+/-H prevalence
														CIMP+	CIMP−		
		CACNA1G	IGF2	NEUROG1	RUNX3	SOCS1	CRABP1	MLH1	p16	MINT1	MINT2	MINT31		CIMP-H	CIMP-L	CIMP-N	
D 1	Samowitz (2005) [13]							+	+	+	+	+		≥2/5	0–1/5		24.6 %
	Lee (2008) [16]							+	+	+	+	+		≥2/5	0–1/5		31.3 %
	Samowitz (2009) [20]							+	+	+	+	+		≥2/5	0–1/5		11.9 %
	Ju (2011) [38]							+	+	+	+	+		≥2/5	0–1/5		24.4 %
D 2	Kalady (2009) [17]	+	+	+	+	+								≥3/5	0–2/5		21.8 %
	Sanchez (2009) [21]	+	+	+	+	+								≥3/5	0–2/5		21.2 %
	Min (2011) [25]	+	+	+	+	+								≥3/5	1–2/5^a^	0/5	13.9 %
	Donada (2013) [40]	+	+	+	+	+								≥3/5	1–2/5^a^	0/5	18.3 %
	Samadder (2013) [30]	+	+	+	+	+								≥3/5	1–2/5^a^	0/5	29.7 %
	Simons (2013) [31]	+	+	+	+	+								≥3/5	0–2/5		Not report
	Cleven (2014) [32]	+	+	+	+	+								≥3/5	0–2/5		Not report
D 3	Bae (2011) [23]	+	+	+	+	+	+	+	+					≥5/8	0–4/8		32.0 %
	Rhee (2012) [26]	+	+	+	+	+	+	+	+					≥5/8	1–4/8^a^	0/8	30.0 %
	Bae (2013) [28]	+	+	+	+	+	+	+	+					≥5/8	1–4/8^a^	0/8	6.4 %
	Kim (2013) [29]	+	+	+	+	+	+	+	+					≥5/8	1–4/8^a^	0/8	29.1 %
	Kim (2009)^b^ [18]	+	+	+	+	+	+	+	+					≥5/8	1–4/8	0/8	11.6 %
D 4	Kim (2009)^c^ [18]	+	+	+	+	+	+	+	+					≥6/8	1–5/8	0/8	7.5 %
	Ogino (2009) [19]	+	+	+	+	+	+	+	+					≥6/8	1–5/8	0/8	19.4 %
	Dahlin (2010) [8]	+	+	+	+	+	+	+	+					≥6/8	1–5/8	0/8	14.2 % 11.4 %^d^
	Dahlin (2011) [24]	+	+	+	+	+	+	+	+					≥6/8	1–5/8	0/8	12.3 %
D 5	Rijnsoever (2002) [12]								+		+		MDR1	≥2/3	0–1/3		32.0 %
D 6	Ward (2003) [37]								+	+	+	+	MINT12	≥4/5	0–3/5		15.4 %
D 7	Kakar (2008) [15]							+	+	+		+	RASSF2, ID4, HIC	≥3/7	0–2/7		23.2 %
	Kakar (2012) [39]							+	+	+		+	RASSF2, ID4, HIC	≥3/7	0–2/7		48.5 %
D 8	Jover (2011) [5]	+		+	+	+		+						≥3/5	0–2/5		29.5 %
D 9	Hokazono (2014) [33]	+							+				ID4, MGMT, TIMP3, TSP1, CDH13, HCAD, GATA5, RSASF1A, HLTF, HRK, KIRREL2, SLC13A5, TSLC1	≥7/15	1–6/15	0/15	18.3 %
D 10	Wang (2014) [36]	+						+	+	+			MGMT, P14ARF	≥3/5	0–2/5		24.0 %
D 11	Barault (2008) [14]							+	+	+	+	+		≥4/5	1–3/5	0/5	16.7 %
D 12	Yagi (2010) [22]	+	+	+	+	+		+	+	+	+	+	MINT17	≥6/11	1–5/11	0/11	11.4 %
D 13	Zlobec (2012) [27]	+		+			+	+	+					≥4/5	1–3/5	0/5	7.1 %
D 14	Li (2014) [34]				+			+	+	+		+	MGMT, APC	≥4/7	1–3/7	0/7	13.1 %

^a^CIMP was classified into three categories, but for analysis of prognosis only two categories were used (CIMP-H vs. CIMP-L/N)

^b^CIMP classification 1 of the study. CIMP-H was defined as ≥5/8 methylated markers, CIMP-L as 1/8 to 4/8 methylated markers, and CIMP-N as 0/8 methylated markers

^c^CIMP classification 2 of the study. CIMP-H was defined as ≥6/8 methylated markers, CIMP-L as 1/8 to 5/8 methylated markers, and CIMP-N as 0/8 methylated markers

^d^CIMP+ or CIMP-H prevalence is 14.2 % in NSHDS study and 11.4 % in CRUMS study. MSHDS and CRUMS are names of two study included in Dahlin et al. study

**Table 2:** CIMP definitions and prevalence in studies on survival after specific colorectal cancer therapies according to CIMP status

Definition	First author (year)	Common CIMP genes											Other CIMP genes	CIMP category			CIMP+/-H prevalence
														CIMP+	CIMP−		
		CACNA1G	IGF2	NEUROG1	RUNX3	SOCS1	CRABP1	MLH1	p16	MINT1	MINT2	MINT31		CIMP-H	CIMP-L	CIMP-N	
D 2	Min (2011) [25]	+	+	+	+	+								≥3/5	1–2/5	0/5	13.9 %
	Donada (2013) [40]	+	+	+	+	+								≥3/5	1–2/5	0/5	18.3 %
	Jo (2012) [44]	+	+	+	+	+								≥3/5	0–2/5		10.0 %
	Shiovitz (2014) [35]	+	+	+	+	+								≥3/5	0–2/5		23.6 %
D 3	Han (2013) [45]	+	+	+	+	+	+	+	+					≥5/8	1–4/8	0/8	7.8 %
D 5	Rijinsoever (2003) [41]								+		+		MDR1	≥2/3	0–1/3		32.5 %
D 8	Jover (2011) [5]	+		+	+	+		+						≥3/5	0–2/5		29.5 %
D 10	Wang (2014) [36]							+	+	+			MGMT, P14^ARF^	≥3/5	<3/5		24.0 %
D 14	Li (2014) [34]				+			+	+	+		+	MGMT, APC	≥4/7	1–3/7	0/7	13.1 %
D 15	Ogino (2007) [42]	+	+	+	+	+	+	+	+	+		+	MGMT, IGFBP3, WRN	≥9/13^a^	1–8/13	0/13	10.0 %
														≥7/13^b^	1–6/13	0/13	16.7 %
D 16	Shen (2007) [43]								+	+		+	P14^ARF^	≥2/4	0–1/4		15.4 %

^a^CIMP classification 1 of the study. CIMP-H was defined as ≥9/13 methylated markers, CIMP-L as 1/13 to 8/13 methylated markers and CIMP-N as 0/13 methylated markers

^b^CIMP classification 2 of the study. CIMP-H was defined as ≥7/13 methylated markers, CIMP-L as 1/13 to 6/13 methylated markers and CIMP-N as 0/13 methylated markers

### CIMP definition and CRC prognosis

As only very few studies investigated the same CIMP definition in association with the same outcome and in similar subgroups, it was not meaningful to perform meta-analyses. According to the available studies, no obvious impact of CIMP definition on the prognostic value of CIMP was found (Tables 3, 4, and 5, Additional file 5: Table S5). Most of the studies reported poorer outcome for CIMP+ patients compared with CIMP− patients, but studies often failed to reach statistical significance due to an insufficient sample size. Some studies just reported univariate results without adjustment for important confounders. Results for CIMP-L were reported for few studies only. Again, the majority of studies showed a trend towards reduced survival which was though not consistent and often not statistically significant. Comparison of the impact of CIMP across studies was hampered by the different definitions of CIMP used. Age and tumor stage were adjusted for in the multivariate analysis of most studies, but BRAF mutation, KRAS mutation, and microsatellite instability (MSI) were assessed as confounding variables only in a few studies. CIMP+ was reported as an independent prognostic for poorer survival in rectal cancer patients compared to CIMP−, both for OS and DFS [20, 28], and was associated with poorer survival also among CRC patients with MSI [8, 23, 26].

**Table 3:** Overall survival among patients with colorectal cancer according to CIMP status

CIMP definition	First author (year)	Subgroup	Subgroup size	Analysis	Survival	HR (95 % CI)		Comparison group	*p* value	Covariates adjusted for
						CIMP+/CIMP-H	CIMP-L			
CRC patients										
D 1	Lee (2008) [16]	All	134	U	OS	1.59 (0.87–2.88)		CIMP−	0.13	No
D 2	Sanchez (2009) [21]	All	391	M	OS	1.56 (0.88–2.78)^a^		CIMP−	0.13	Age, sex, stage, location, MSI^b^
D 6	Ward (2003) [37]	All	609	U	OS	1.30 (0.70–2.20)		CIMP−	0.37	No
D 7	Kakar (2012) [39]	All	33	U	OS	1.19 (0.51–2.58)		CIMP−	0.69	No
D 14	Li (2014) [34]	All	282	M	OS	2.31 (1.02–5.24)		CIMP−	0.04	Age, stage, location, differentiation
D 2	Samadder (2013) [30]	All	563	M	OS	1.12 (0.81–1.55)	0.86 (0.60–1.23)	CIMP-N	0.60	Age, stage, grade, location^c^
D 3	Kim (2009) [18]	All	320	U	OS	1.81 (0.91–3.66)	1.33 (0.78–2.27)	CIMP-N	0.24	No
D 4	Kim (2009) [18]	All	320	U	OS	2.46 (1.16–5.19)	1.30 (0.76–2.22)	CIMP-N	0.05	No
D 14	Li (2014) [34]	All	282	M	OS	3.06 (1.19–7.89)	0.95 (0.60–1.52)	CIMP-N	0.02	Age, stage, location, differentiation
CRC patients by cancer stages										
D 14	Li (2014) [34]	I–III	149	M	OS	0.52 (0.12–2.22)		CIMP−	0.38	Age, stage, location, differentiation
D 6	Ward (2003) [37]	I–III	476	U	OS	1.20 (0.60–2.80)		CIMP−	0.60	No
D 11	Barault (2008) [14]	I–II, colon, MSS	246	M	OS^d^	2.90 (1.53–5.49)	1.85 (1.37–2.51)	CIMP-N	<0.01	Age, stage, BRAF, KRAS
D 2	Donada (2013) [40]	II, colon	120	M	OS	0.60		CIMP−	0.30	All variables^e^
D 14	Li (2014) [34]	III–IV	129	M	OS	1.75 (0.95–3.23)		CIMP−	0.07	Age, stage, location, differentiation
CRC patients by location of CRC										
D 3	Bae (2013) [28]	Proximal	165	M	OS	0.84 (0.42–1.69)		CIMP−	0.62	Stage^,^ differentiation^f^
D 3	Bae (2013) [28]	Distal	327	M	OS	1.35 (0.47–3.90)		CIMP−	0.58	Stage^,^ differentiation^f^
D 4	Ogino (2009) [19]	Colon	649	M	OS	0.78 (0.47–1.29)	1.01 (0.77–1.33)	CIMP-N		Age, sex, stage, BRAF, KRAS, MSI^g^
D 2	Donada (2013) [40]	Colon, II	120	M	OS	0.60		CIMP−	0.30	All variables^e^
D 1	Samowitz (2005) [13]	Colon, MSS	803	M	OS	0.88 (0.66–1.18)		CIMP−		Age, stage, location, BRAF
D 11	Barault (2008) [14]	Colon, MSS, I–II	246	M	OS^d^	2.90 (1.53–5.49)	1.85 (1.37–2.51)	CIMP−	<0.01	Age, stage, BRAF, KRAS
D 3	Bae (2013) [28]	Rectal	242	M	OS	4.13 (1.27–13.46)		CIMP−	0.02	Stage^,^ differentiation^f^
CRC patients by microsatellite instability										
D 1	Lee (2008) [16]	MSS	115	U	OS	1.96 (1.06–3.61)		CIMP−	0.03	No
D 6	Ward (2003) [37]	MSS	547	M	OS	2.10 (1.10–4.00)		CIMP−	0.02	Stage, vascular space invasion
D 7	Kakar (2008) [15]	MSS	69	M	OS	0.86 (0.35–2.13)		CIMP−	0.70	Age, sex, stage, LOH, BRAF
D 6	Ward (2003) [37]	MSS, I–III	464	M	OS	2.40 (0.94–6.00)		CIMP−	0.06	Stage, vascular space invasion
D 1	Samowitz (2005) [13]	MSS, colon	803	M	OS	0.88 (0.66–1.18)		CIMP−		Age, stage, location, BRAF
D 11	Barault (2008) [14]	MSS, colon, I–II	246	M	OS^d^	2.90 (1.53–5.49)	1.85 (1.37–2.51)	CIMP-N	<0.01	Age, stage, BRAF, KRAS
CRC patients by microsatellite instability										
D 3	Bae (2011) [23]	MSI	169	M	OS	2.81 (1.08–7.27)		CIMP−	0.03	Age, stage, BRAF, differentiation^h^
D 3	Rhee (2012) [26]	MSI	207	M	OS	3.05 (1.07–8.73)		CIMP−	0.04	Age, stage, location, BRAF/KRAS^i^

*CI* confidence interval, *HR* hazard ratio, *M* multivariate analysis, *OS* overall survival, *U* univariate analysis

^a^The original data of HR (95 % CI) was 0.64 (0.36–1.14) derived from CIMP− compared with CIMP+

^b^Other covariates included in the multivariate analysis are differentiation and chemotherapy

^c^Other covariates included in the multivariate analysis are chemotherapy and radiation therapy

^d^Relative survival ratio of observed survival rate to the expected survival rate in a population with similar sex and age distribution derived from local mortality

^e^Original paper mentioned the multivariate analysis including all clinical, pathological, and molecular variables

^f^Other covariates included in the multivariate analysis are adjuvant chemotherapy

^g^Other covariates included in the multivariate analysis are year of diagnosis, location, and tumor grade

^h^Other covariates included in the multivariate analysis are Crohn’s-like lymphoid reaction, peritumoral lymphocytic reaction, and postoperative chemotherapy

^i^Other covariates included in the multivariate analysis are grade, gross type and Crohn’s-like lymphoid reaction, and peritumoral lymphocytic reaction

**Table 4:** Disease-specific survival among patients with colorectal cancer according to CIMP status

CIMP definition	First author (year)	Subgroup	Subgroup size	Analysis	Survival	HR (95 % CI)		Comparison group	*p* value	Covariates adjusted for
						CIMP+/CIMP-H	CIMP-L			
CRC patients										
D 2	Simons (2013) [31]	All	27	M	DSS	3.67 (1.70–7.91)^a^		CIMP−		Age, sex, stage, location^b^
D 2	Samadder (2013) [30]	All	563	M	DSS	1.06 (0.65–1.35)	1.19 (0.72–1.97)	CIMP-N	0.74	Age, stage, grade, location^c^
D 4	Dahlin (2010) [8]	All (NSHDS)	190	M	DSS	1.84 (0.87–3.89)	2.01 (1.20–3.37)	CIMP-N		Age, sex, stage, location^d^
D 4	Dahlin (2010) [8]	All (CRUMS)	414	M	DSS	1.10 (0.59–2.03)	1.48 (1.00–2.22)	CIMP-N		Age, sex, stage, location^d^
D 4	Dahlin (2011) [24]	All	484	M	DSS	1.09 (0.59–2.03)	1.47 (0.98–2.20)	CIMP-N		Age, sex, stage, location^d^
CRC patients by microsatellite instability										
D 1	Samowitz (2005) [13]	MSS, colon	803	M	DSS	0.97 (0.70–1.36)		CIMP−		Age, stage, location, BRAF
D 4	Dahlin (2010) [8]	MSS (NSHDS)	166	M	DSS	3.05 (1.40–6.63)	1.89 (1.12–3.21)	CIMP-N		Age, sex, stage, location^d^
D 4	Dahlin (2010) [8]	MSS (CRUMS)	338	M	DSS	1.38 (0.62–3.07)	1.45 (0.95–2.23)	CIMP-N		Age, sex, stage, location^d^
D 4	Dahlin (2010) [8]	MSI (CRUMS)	62	M	DSS	1.23 (0.13–11.23)	3.87 (0.46–32.39)	CIMP-N		Age, sex, stage, location^d^
CRC patients by location of CRC										
D 4	Ogino (2009) [19]	Colon	649	M	DSS	0.44 (0.22–0.88)	0.78 (0.54–1.11)	CIMP-N		Age, stage, BRAF, KRAS, MSI^e^
D 1	Samowitz (2005) [13]	MSS, colon	803	M	DSS	0.97 (0.70–1.36)		CIMP−		Age, stage, location, BRAF

*CI* confidence interval, *CRUMS and NSHDS* are the names of two studies included in Dahlin et al. study, *DSS* disease-specific survival, *HR* hazard ratio, *M* multivariate analysis, *U* univariate analysis

^a^Data come from early follow-up (≤2 year). Data of late follow-up (>2 year) was 1.41 (0.43–4.57)

^b^Other covariates included in the multivariate analysis are differentiation grade and initial adjuvant therapy

^c^Other covariates included in the multivariate analysis are chemotherapy and radiation therapy

^d^Other covariate included in the multivariate analysis is adjuvant chemotherapy

^e^Other covariates included in the multivariate analysis are year of diagnosis, sex, site, and tumor grade

**Table 5:** Disease-free survival or recurrence-free survival among patients with colorectal cancer according to CIMP status

CIMP definition	First author (year)	Subgroup	Subgroup size	Analysis	Survival	HR (95 % CI)		Comparison group	*p* value	Covariates adjusted for
						CIMP+/ CIMP-H	CIMP-L			
CRC patients										
D 2	Kalady (2009) [17]	All	357	M	RFS	2.08 (0.65–6.65)^a^		CIMP−	0.21	Age, sex, stage, MSI^b^
D 9	Hokazono (2014) [33]	All	104	M	RFS	0.29 (0.02–1.42)		CIMP−	0.15	Not report
CRC patients by cancer stages										
D 9	Hokazono (2014) [33]	I–II	50	M	RFS	0.01 (0.00–2.28)		CIMP−		Not report
D 1	Ju (2011) [38]	I–III	53	M	RFS	1.05 (0.33–3.39)		CIMP−	0.93	Age, sex, stage
D 2	Min (2011) [25]	I–III	124	M	RFS	0.81 (0.21–3.14)		CIMP−		Age, sex, stage, BRAF, KRAS, MGMT^b^
D 6	Ward (2003) [37]	I–III	476	U	RFS	0.90 (0.50–2.00)		CIMP−	0.94	No
D 8	Jover (2011) [5]	II–III	196	M	DFS	1.20 (0.80–2.00)		CIMP−	0.40	Age, stage, adjuvant chemotherapy
D 10	Wang (2014) [36]	II–III	50	M	DFS	2.94 (1.19–7.22)		CIMP−	0.02	Stage, location
D 2	Donada (2013) [40]	II, colon	120	M	DFS	0.58		CIMP−	0.20	All variables^c^
D 9	Hokazono (2014) [33]	III	39	M	RFS	0.45 (0.01–2.23)		CIMP−		Not report
CRC patients by location of CRC										
D 3	Bae (2013) [28]	Proximal	165	M	DFS	1.00 (0.53–1.88)		CIMP−	0.99	Stage^c^
D 3	Bae (2013) [28]	Distal	327	M	DFS	1.31 (0.51–3.36)		CIMP−	0.58	Stage^c^
D 2	Donada (2013) [40]	Colon, II	120	M	DFS	0.58		CIMP−	0.20	All variables^c^
D 1	Samowitz (2009) [20]	Rectal	990	M	DFS	1.32 (0.88–1.97)		CIMP−		Age, stage, BRAF, MSI, KRAS, TP53^d^
D 3	Bae (2013) [28]	Rectal	242	M	DFS	2.90 (1.04–8.08)		CIMP−	0.04	Stage^c^
CRC patients by microsatellite instability										
D 3	Kim (2013) [29]	MSI	220	M	DFS	2.25 (1.11–4.57)		CIMP−	0.03	Age, stage, differentiation, BRAF^e^

*CI* confidence interval, *DFS* disease-free survival, *HR* hazard ratio, *M* multivariate analysis, *RFS* recurrence-free survival, *U* univariate analysis

^a^The original data of HR (95 % CI) was 0.48 (0.15–1.53) derived from CIMP− compared with CIMP+

^b^Other covariates included in the multivariate analysis are: differentiation, chemotherapy

^c^Original paper mentioned the multivariate analysis including all clinical, pathological and molecular variables

^d^Multivariate analysis adjusted BRAF, MSI, KRAS, and TP53, in addition to age and stage, the result adjusted for only age and stage was 1.45 (1.02–2.07)

^e^Other covariates included in the multivariate analysis are Crohn’s-like lymphoid reaction and peritumoral lymphocytic reaction

### Survival after specific CRC therapies according to CIMP status

The limited number of studies on survival after specific CRC therapies did not show a clear pattern of effect modification of chemotherapy success by CIMP status (Table 6). Among patients with CIMP+, three studies showed no statistically significant differences on survival for patients who were treated with surgery and chemotherapy compared with surgery alone. Results were inconsistent among patients with CIMP−. Shiovitz et al. [35] reported potentially enhanced survival after surgery + FU + LV + IFL therapy compared to patients receiving surgery + FU + LV therapy only among CIMP+ patients (HR, 0.62; 95 % CI, 0.37–1.05), while CIMP− patients had a lower DFS with the combination therapy (HR, 1.38; 95 % CI, 1.00–1.89).

**Table 6:** Survival of colorectal cancer patients treated with surgery and chemotherapy compared with patients treated with surgery alone

CIMP group	CIMP definition	Study	Group size	Analysis	Survival	HR (95 % CI)	*p* value
CIMP+	D 14	Li (2014) [34]	37	M	OS	0.71 (0.20–2.54)	0.60
	D 2	Shiovitz (2014)^a^ [35]	145	M	OS	0.62 (0.37–1.05)^b^	0.07
	D 8	Jover (2011) [5]	89	M	DFS	0.80 (0.30–2.00)	0.60
CIMP−	D 14	Li (2014) [34]	245	M	OS	1.09 (0.65–1.82)	0.75
	D 2	Shiovitz (2014)^a^ [35]	470	M	OS	1.38 (1.00–1.89)	0.05
	D 5	Rijinsoever (2003) [41]	139	M	DSS	0.96 (0.62–1.49)	0.86
	D 8	Jover (2011) [5]	213	M	DFS	0.40 (0.20–0.60)	<0.01

*CI* confidence interval, *HR* hazard ratio

^a^Results comparing patients treated with surgery + LV + FU + IFL with patients treated with surgery + LV + FU

^b^HR of surgery + chemotherapy compared with surgery alone in CIMP+ group was not reported directly in the study and was calculated according to 95 % CI

## Discussion

Based on the results of this review, predominantly poorer survival was observed in patients with CIMP+ or CIMP-H. Regarding CIMP definitions, no obvious difference could be observed in the prognostic value of the 16 different CIMP panels due to the high heterogeneity of CIMP definitions, subgroups, and outcomes investigated. A plausible reason might be that CIMP in most studies contains overlapping gene markers to various extents. Most of the studies chose commonly used CIMP-related gene markers to define CIMP except for the study by Hokazono et al. [33]. Among the 15 included in CIMP definitions, MLH1, MINT1, CACNA1G, and p16 were the most commonly used (Table 1). Other genes like MGMT and ID4 were only included by a few studies.

Furthermore, categorization of CIMP in two categories (CIMP+ vs. CIMP−) or three categories (CIMP-H vs. CIMP-L or CIMP-0) may result in different associations with survival after CRC. CIMP-L represents a subclass of CRCs with intermediate methylation level and this intermediate methylation subclass shows distinct features compared with CIMP-H or CIMP-0, respectively. CIMP-H CRC shows associations with MSI, more frequent BRAF mutation, and less KRAS and TP53 mutations, whereas CIMP-L CRCs are associated with KRAS mutation. CIMP-0 is characterized by frequent TP53 mutation [22, 50–52]. Another source of heterogeneity is threshold for CIMP-H. In the study by Kim et al. [18], CIMP-H defined by six or more methylated loci (definition 4) showed a statistically significant and a somewhat stronger association with poorer survival compared to a cutoff of five or more methylated loci (definition 3). Lee and colleagues also found that higher cutoff for CIMP+ could better separate the survival curve of CIMP+ and CIMP− patients among MSI CRC [53].

Also, the use of different laboratory methods and criteria for threshold of methylation may influence findings. MSP is a simple, sensitive, and specific method for determining the methylation status of virtually any CpG-rich region [46]. MethyLight, compared with MSP, is not only a sensitive and accurate method to detect methylation but also a quantitative real-time PCR analysis technique with high-throughput capability [47]. Quantitative DNA methylation analysis is used because it was found that low levels of DNA methylation do not generally silence gene expression [54, 55]. Moreover, the cutoff for gene methylation used in each method also varied between studies. Besides, DNA samples obtained from tissue preserved in different ways might cause inconsistent methylation levels. A previous study have shown that DNA samples obtained from FFPE tissue can provide accurate and reproducible results in DNA methylation analyses by using Infinium HumanMethylation450 BeadChip (HM-450 K) assays or nested MSP [56]. However, there is limited evidence on the effect of different laboratory methods and no obvious differences in outcomes according to laboratory method were observed in this review with respect to the prognostic value of CIMP. Further research should address the potential role of laboratory methods for the prognostic value of CIMP definition in more detail.

Besides the difference in CIMP definition and laboratory methods, failure in controlling important confounders in the respective studies may cause the observed discrepancies in the CIMP literature. Patient and clinical factors that are associated with both CIMP and CRC prognosis, such as age, tumor location, and stage, were adjusted for in most but not in all of the included studies. CIMP-high tumors are more often present in women than in men. However, gender was only adjusted for in a few studies and none of the included studies reported prognostic implications of CIMP according to gender.

Patients with MSI CRC were shown to have better prognosis than patients with microsatellite stability (MSS) CRC [17]. Ward et al. [37] even found that the adverse prognostic effect of CIMP could be reversed by MSI. However, in studies by Bea et al. and by Kim et al. [23, 29], CIMP+ was found to be associated with poorer survival among patients with MSI CRC. In addition, BRAF mutation was shown to be a predictor of poorer outcome in patients with MSS CRC [15]. In the study by Samowitz et al. [13], the association between CIMP+ and worse outcomes of MSS CRC disappeared after adjusting for BRAF. The impact of other genetic factors, such as KRAS mutation, on the prognosis of CRC is also not consistent [20, 21, 29]. Sporadic MSI-H and BRAF mutations frequently occur in a subset of CIMP tumors; however, they were only assessed in a few studies but may play a role in the association between CIMP and CRC prognosis. Some researchers even suggested new major subtypes of CRC that combine two or even four of these factors which might provide a new approach to investigate and understand the relationship between genetic markers and the prognosis of CRC [57].

Results regarding to potential role of CIMP in predicting success of specific therapies were inconsistent. In one study [35], addition of IFL to therapy with surgery + LV + FU was associated with tentatively enhanced survival among CIMP+ patients and tentatively worse survival among CIMP− patients. More and larger studies are needed to find out if CIMP is a relevant molecular biomarker for adjuvant therapy decision-making in CRC.

The main limitation of our review was the lack of studies investigating the same CIMP definitions. Moreover, the sample sizes of included studies were all less than 1000 and mostly less than 400 cases with four of the studies only including less than 100 cases. The statistical power in these studies became even weaker when analyzing subgroups. Thus, more studies in larger patient populations are needed.

## Conclusions

In summary, no clear differences could be observed regarding the association of CIMP and CRC prognosis that are based on the different CIMP definitions due to the high heterogeneity of CIMP definitions, subgroups, and outcomes investigated. Although CIMP marker panel, CIMP grouping, and threshold of CIMP+ or CIMP-H may have potential impact on the prognostic value of CIMP among CRC patients, evidence on their specific impact is still limited. Comparative analyses of different CIMP panels in the same large study populations are needed to further clarify the role of CIMP definitions and to find out how methylation information can best be used to predict CRC prognosis and response to specific CRC therapies.

## Additional files

Additional file 1: Table S1. General information of studies investigating survival after colorectal cancer according to CIMP status. (DOCX 28 kb) Additional file 2: Table S2. General information of studies investigating therapy response after colorectal cancer according to CIMP status. (DOCX 22 kb) Additional file 3: Table S3. Method used for methylation analysis in studies on colorectal cancer survival according to CIMP status. (DOCX 24 kb) Additional file 4: Table S4. Method used for methylation analysis in studies investigating therapy response after colorectal according to CIMP status. (DOCX 20 kb) Additional file 5: Table S5. Results of studies on survival after colorectal cancer according to CIMP status reporting a *p* value only. (DOCX 22 kb)

## Abbreviations

COBRA: combined bisulfite restriction analysis
CI: confidence interval
CIMP: CpG island methylator phenotype
CIMP+: CIMP-positive
CIMP−: CIMP-negative
CIMP-H: CIMP-high
CIMP-L: CIMP-low
CIMP-N: CIMP-negative
CRC: colorectal cancer
DFS: disease-free survival
DSS: disease-specific survival
FFPE: formalin-fixed and paraffin-embedded
FF: fresh-frozen
HR: hazard ratio
M: multivariate analysis
MS-HRM: methylation-sensitive high-resolution melting
MSI: microsatellite instability
MSP: methylation-specific PCR
MSS: microsatellite stability
OS: overall survival
RFS: recurrence-free survival
