# Supplementary material for: Different definitions of CpG island methylator phenotype and outcomes of colorectal cancer: a systematic review
Source: Clin Epigenetics. 2016 Mar 2;8:25. doi: 10.1186/s13148-016-0191-8 (PMC4776403; doi:10.1186/s13148-016-0191-8)
Supplement: Additional file 1: Table S1. — General information of studies investigating survival after colorectal cancer according to CIMP status. (DOCX 28 kb) [file 13148_2016_191_MOESM1_ESM.docx]

Additional file 1: Table S1. General information of studies investigating survival after colorectal cancer according to CIMP status.

| **First author (year)** | **Country** | **Population** | **Size** | **Age (years)** | **Sex**  **(males)** | **Follow-up time**  **(years)** | **Outcome** | | | |  |
| --- | --- | --- | --- | --- | --- | --- | --- | --- | --- | --- | --- |
|  |  |  |  |  |  |  | OS | DSS | DFS | RFS | |
| Rijnsoever (2002) [12] | Australia | Stage II/III CRC | 275 | 52.3%>71 ^a^ | 50.2% | 5.0 years^b^ | + |  |  |  | |
| Ward (2003) [37] | Australia | CRC | 605 | 68.3 (mean) | 53.4% | 2.7 (median) | + |  |  |  | |
| Samowitz (2005) [13] | USA | Colon cancer | 886 | 36.9%>71~79 | 53.4% |  | + | + |  |  | |
| Barault (2008) [14] | France | Colon cancer | 582 | 41.6%>75 | 57.2% | 5.0 years^b^ |  |  |  |  | |
| Kakar (2008) [15] | USA | CRC | 83 | 71.2%>60 | 56.5% | 5.0 years^b^ | + |  |  |  | |
| Lee (2008) [16] | Korea | CRC | 134 | 57.5%≥60 | 60.4% | 5.0 years^b^ | + |  |  |  | |
| Kalady (2009) [17] | USA | CRC | 357 | 66.9 (mean) | 51.4% | 3.3 (mean) |  |  | + | + | |
| Kim (2009) [18] | Korea | CRC | 320 | 51.4%≥56 | 58.4% | 5.3 (mean) | + |  |  |  | |
| Ogino (2009) [19] | USA | Colon cancer | 649 | 66.5 (mean) | 44.0% |  | + | + |  |  | |
| Samowitz (2009) [20] | USA | Rectal cancer | 990 |  |  | 5.7 (median) | + |  |  |  | |
| Sanchez (2009) [21] | USA | CRC | 391 | 66.7 (mean) | 55.0% | 3.2 (mean) | + |  |  |  | |
| Dahlin (2010) [8] | Sweden | CRC, NSHDS^c^ | 190 | 63 (median) | 43.2% |  |  | + |  |  | |
|  |  | CRC, CRUMS^c^ | 414 | 73 (median) | 56.3% |  |  | + |  |  | |
| Yagi (2010) [22] | Japan | CRC | 149 | 62.8 (mean) | 57.0% | 4.1 (mean) | + |  |  |  | |
| Bae (2011) [23] | Korea | MSI CRC | 72^d^  97^d^ |  |  | 3.2 (mean)  4.4 (mean) | + |  |  |  | |
| Dahlin (2011) [24] | Sweden | CRC | 484 | 71 (median) | 55.0% | 5.0 years^b^ |  | + |  |  | |
| Jover (2011) [5] | Spain | CRC | 302 |  | 86.2% | 4.2 (median) |  |  | + |  | |
| Ju (2011) [38] | Japan | CRC | 78 |  | 65.4% | 1.8 (median） |  |  | + |  | |
| Min (2011) [25] | Korea | Stage I-III CRC | 124 |  | 57.3% | 3.7 (median) |  |  |  | + | |
| Kakar (2012) [39] | USA | Signet ring call cancer | 33 | 39.4%>60 | 72.7% | 5.0 years^b^ | + |  |  |  | |
| Rhee (2012) [26] | Korea | MSI CRC | 207 | 51.7%>55 | 60.9% | 3.8 (mean) | + |  |  |  | |

Supplementary Table 1 continued.

| **First author (year)** | **Country** | **Population** | **Size** | **Age (years)** | **Sex**  **(males)** | **Follow-up time**  **(years)** | **Outcome** | | | |  |
| --- | --- | --- | --- | --- | --- | --- | --- | --- | --- | --- | --- |
|  |  |  |  |  |  |  | OS | DSS | DFS | RFS | |
| Zlobec (2012) [27] | Switzerland | CRC | 337 | 69.9 (mean) | 46.3% | 5.0 years^b^ |  | + |  |  | |
| Bae (2013) [28] | Korea | CRC | 734 | 62 (median) | 60.8% | 4.7 (mean) | + |  | + |  | |
| Donada (2013) [40] | Italy | Stage II colon cancer | 120 | 67.6 (mean) | 47.5% | 9.4 (median) | + |  | + |  | |
| Kim (2013) [29] | Korea | MSI CRC | 220 | 51.4%>56 | 60.5% | 5.0 years^b^ |  |  | + |  | |
| Samadder (2013) [30] | USA | CRC | 563 | 73.9 (mean) |  |  | + | + |  |  | |
| Simons (2013) [31] | Netherlands | CRC | 27 | 67.6 (mean) | 55.6% | 8.4 (median) |  | + |  |  | |
| Cleven (2014) [32] | Netherlands | CRC | 173^e^  569^e^ | 67.8 (mean)  63.1 (mean) | 47.0%  53.3% | 4.8 (median)  8.9 (median) |  | + |  |  | |
| Hokazono (2014) [33] | Japan | CRC | 104 | 63.4 (mean) | 49.0% | 5.0 (median) | + |  | + |  | |
| Li (2014) [34] | China | CRC | 282 | 58.8 (mean) | 58.5% | 4.4 (median) | + |  |  |  | |
| Wang (2014) [36] | China | Stage II/III CRC | 50 |  | 46.0% | 5.0 years^b^ | + |  | + |  | |

a: there are 52.3% of the populations with the age more than 71 years old in this study.

b: overall follow-up time according to figures without specific data in text.

c: Name of two independent studies included in Dahlin et al. study.

d: data come from two independent studies.

e: data come from study population (n=173) and validation population (n=569).
