# Supplementary material for: Different definitions of CpG island methylator phenotype and outcomes of colorectal cancer: a systematic review
Source: Clin Epigenetics. 2016 Mar 2;8:25. doi: 10.1186/s13148-016-0191-8 (PMC4776403; doi:10.1186/s13148-016-0191-8)
Supplement: Additional file 2: Table S2. — General information of studies investigating therapy response after colorectal cancer according to CIMP status. (DOCX 22 kb) [file 13148_2016_191_MOESM2_ESM.docx]

Additional file 2: Table S2. General information of studies investigating therapy response after colorectal cancer according to CIMP status.

| **First author (year)** | **Country** | **Population** | **Size** | **Age (years)** | **Sex (males)** | **Follow-up (years)** | **Therapy** |
| --- | --- | --- | --- | --- | --- | --- | --- |
| Rijinsoever (2003) [41] | Australia | Stage III CRC | 206 |  | 64.1% | 3.3 (median) | Surgery vs. surgery+ 5FU |
| Ogino (2007) [42] | USA | MSS metastatic CRC | 30 | 54.9 (mean) | 71.0% | 2.8 (median) | Chemotherapy^a^ |
| Shen (2007) [43] | Pennsylvania | Stage IV or recurrent CRC | 188 | 50%>65^b^ | 65.9% | 5.0 years^c^ | Chemotherapy^d^ |
| Jover (2011) [5] | Spain | Stage II and III CRC | 302 |  | 86.2% | 4.6 (median) | Surgery vs. surgery+ 5FU |
| Min (2011) [25] | Korea | Stage II and III CRC | 124 |  | 57.3% | 3.7 (median) | Surgery vs. surgery+ 5FU |
| Jo (2012) [44] | Germany | Stage II and III Rectal cancer | 150 |  | 71.0% | 5.0 years^c^ | Surgery+ RCT |
| Donada (2013) [40] | Italy | Stage II colon cancer | 120 | 67.6 (mean) | 47.5% | 9.4 (median) | Surgery vs. surgery+ 5FU |
| Han (2013) [45] | Korea | Stage II and III Rectal cancer | 322 | 61.0 (median) | 59.6% | 3.3 (median) | Surgery+ FOLFOX |
| Li (2014) [34] | China | CRC | 282 | 58.8 (mean) | 58.5% | 4.4 (median) | Surgery vs. surgery+ 5FU |
| Shiovitz (2014) [35] | USA | Stage III CRC | 615 | 63.0 (median) | 55.0% | 4.8 (median) | Surgery+ LV+ FU vs. Surgery+ LV+ FU+ IFL |
| Wang (2014) [36] | China | Stage II/III CRC | 50 |  | 46.0% | 5.0 years^c^ | Surgery vs. surgery+ chemotherapy^e^ |

Abbreviations: FOLFOX, leucovorin, 5-fluorouracil and oxalplatin; FU, fluorouracil; 5FU, 5-fluorouracil-based adjuvant chemotherapy; IFL, irinotecan; LV, leucovorin; RCT, radiochemotherapy.

a: combined therapy with 5-fluorouracil, irinotecan, leucovorin and gefitnib.

b: there are 50% of the populations with the age more than 65 years old in this study.

c: overall follow-up time according to figures without specific data in the text.

d: treated with 5-fluorouracil or 5-fluorouracil combined with oral leucovorin, i.v.leucovorin, IFNα-2a or N-phosphonoacetyl-L-aspartic acid.

e: adjuvant chemotherapy using either standard m FOLFOX6 or CapeOX regimen.
