# Supplementary material for: Different definitions of CpG island methylator phenotype and outcomes of colorectal cancer: a systematic review
Source: Clin Epigenetics. 2016 Mar 2;8:25. doi: 10.1186/s13148-016-0191-8 (PMC4776403; doi:10.1186/s13148-016-0191-8)
Supplement: Additional file 3: Table S3. — Method used for methylation analysis in studies on colorectal cancer survival according to CIMP status. (DOCX 24 kb) [file 13148_2016_191_MOESM3_ESM.docx]

Additional file 3: Table S3. Method used for methylation analysis in studies on colorectal cancer survival according to CIMP status.

| **Definition** | **First author (year)** | **Method** | **Cutoff** | **DNA sample tissue** |
| --- | --- | --- | --- | --- |
| D 1 | Samowitz (2005) [13] | MSP |  | Paraffin-embedded tissue |
|  | Lee (2008) [16] | MSP |  | Paraffin-embedded tissue |
|  | Samowitz (2009) [20] | MSP |  | Paraffin-embedded tissue |
|  | Ju (2011) [38] | MSP |  | FF tissue |
| D 2 | Kalady (2009) [17] | MethyLight | PMR>10 | FF tissue |
|  | Sanchez (2009) [21] | MethyLight | PMR>10 | FF tissue |
|  | Min (2011) [25] | MethyLight |  | Paraffin-embedded tissue |
|  | Donada (2013) [40] | MSP |  | Paraffin-embedded tissue |
|  | Samadder (2013) [30] | MethyLight | PMR>10 | Paraffin-embedded tissue |
|  | Simons (2013) [31] | MSP |  | Paraffin-embedded tissue |
|  | Cleven (2014) [32] | MSP |  | Not reported |
| D 3 | Bae (2011) [23] | MethyLight | PMR>4 | Paraffin-embedded tissue |
|  | Rhee (2012) [26] | MethyLight | PMR>4 | Paraffin-embedded tissue |
|  | Bae (2013) [28] | MethyLight | PMR>4 | Paraffin-embedded tissue |
|  | Kim (2013) [29] | MethyLight | PMR>4 | Paraffin-embedded tissue |
| D 4 | Kim (2009) [18] | MethyLight | PMR>4 | Paraffin-embedded tissue |
|  | Ogino (2009) [19] | MethyLight |  | Paraffin-embedded tissue |
|  | Dahlin (2010) [8] | MethyLight | PMR>10 | Paraffin-embedded tissue |
|  | Dahlin (2011) [24] | MethyLight | PMR>10 | Paraffin-embedded tissue |
| D 5 | Rijnsoever (2002) [12] | MSP |  | FF tissue |
| D 6 | Ward (2003) [37] | MSP |  | FF tissue |
| D 7 | Kakar (2008) [15] | MSP |  | Paraffin-embedded tissue |
|  | Kakar (2012) [39] | MSP |  | Paraffin-embedded tissue |
| D 8 | Jover (2011) [5] | Pyrosequencing | 5%^a^ | Not reported |
| D 9 | Hokazono (2014) [33] | MSP |  | FF tissue |
| D 10 | Wang (2014) [36] | MSP |  | FF tissue |
| D 11 | Barault (2008) [14] | MSP |  | Not reported |
| D 12 | Yagi (2010) [22] | MassARRAY |  | FF tissue |
| D 13 | Zlobec (2012) [27] | Pyrosequencing | 30%^a^ | Paraffin-embedded tissue |
| D 14 | Li (2014) [36] | MS-HRM | 5%^a^ | Not reported |

Abbreviations: FF, fresh-frozen; MS-HRM, methylation sensitive high resolution melting; MSP, methylation-specific PCR; PMR, the percentage of methylated reference. PMR=100*[(methylated reaction/ALU) sample /(methylated reaction/ALU) M.SssI-reference].

a: the mean percentage more than 5% in the studies by Jover et al. and Li et al. or 30% in the study by Bae et al..
