# Supplementary material for: Different definitions of CpG island methylator phenotype and outcomes of colorectal cancer: a systematic review
Source: Clin Epigenetics. 2016 Mar 2;8:25. doi: 10.1186/s13148-016-0191-8 (PMC4776403; doi:10.1186/s13148-016-0191-8)
Supplement: Additional file 4: Table S4. — Method used for methylation analysis in studies investigating therapy response after colorectal according to CIMP status. (DOCX 20 kb) [file 13148_2016_191_MOESM4_ESM.docx]

Additional file 4: Table S4. Method used for methylation analysis in studies investigating therapy response after colorectal according to CIMP status.

| **Definition** | **First author (year)** | **Method** | **Cutoff** | **DNA sample tissue** |
| --- | --- | --- | --- | --- |
| D 2 | Min (2011) [25] | MethyLight |  | Paraffin-embedded tissue |
|  | Jo (2012) [44] | MSP |  | Paraffin-embedded tissue |
|  | Donada (2013) [40] | MSP |  | Paraffin-embedded tissue |
|  | Shiovitz (2014) [35] | MethyLight | PMR >4 | Paraffin-embedded tissue |
| D 3 | Han (2013) [45] | MethyLight | PMR >4 | Not reported |
| D 5 | Rijinsoever (2003) [41] | MSP |  | Paraffin-embedded tissue |
| D 8 | Jover (2011) [5] | MSP | 5%^a^ | Not report |
| D 10 | Wang (2014) [36] | MSP |  | FF tissue |
| D 14 | Li (2014) [34] | MS-HRM | 5%^a^ | Not reported |
| D 15 | Ogino (2007) [42] | MethyLight | PMR >4 | Paraffin-embedded tissue |
| D 16 | Shen (2007) [43] | COBRA | 15%^a^ | Paraffin-embedded tissue |

Abbreviations: COBRA, combined bisulfite restriction analysis; FF, fresh-frozen; MS-HRM, methylation sensitive high resolution melting; MSP, methylation-specific PCR; PMR, the percentage of methylated reference. PMR=100*[(methylated reaction/ALU) sample / (methylated reaction/ALU) M.SssI-reference].

a: the mean percentage more than 5%.
