# Supplementary material for: Different definitions of CpG island methylator phenotype and outcomes of colorectal cancer: a systematic review
Source: Clin Epigenetics. 2016 Mar 2;8:25. doi: 10.1186/s13148-016-0191-8 (PMC4776403; doi:10.1186/s13148-016-0191-8)
Supplement: Additional file 5: Table S5. — Results of studies on survival after colorectal cancer according to CIMP status reporting a p value only. (DOCX 22 kb) [file 13148_2016_191_MOESM5_ESM.docx]

Additional file 5: Table S5. Results of studies on survival after colorectal cancer according to CIMP status reporting a P value only.

| **CIMP definition** | **First author (year)** | **Subgroup** | **Analysis** | **Survival** | **Subgroup size** | **Comparison group** | **P value** |
| --- | --- | --- | --- | --- | --- | --- | --- |
| D 3 | Bae (2013) [28] | All | M | OS | 734 | CIMP- | 0.45 |
| D 5 | Rijnsoever (2002) [12] | II/III | M | OS | 275 | CIMP- | >0.05 |
| D 10 | Wang (2014) [36] | II/III | U | OS | 50 | CIMP- | 0.35 |
| D 1 | Samowitz (2005) [13] | Colon | U | OS | 886 | CIMP- | 0.12 |
| D 1 | Samowitz (2005) [13] | Colon, MSI | U | OS | 83 | CIMP- | 0.77 |
| D 2 | Cleven (2014) [32] | All |  | DSS | 173, 569^a^ | CIMP- | 3.81, 0.04^b^ |
| D 8 | Jover (2011) [5] | All | U | DFS | 302 | CIMP- | 0.60 |

Abbreviations: CI, confidence interval; DFS, disease-free survival; DSS, disease-specific survival; HR, hazard ratio; M, multivariate analysis; OS, overall survival; U, indicate univariate analysis;

a: group size of study population (n=173) and validation population (n=569).

b: P value of study population (P=3.81) and validation population (P=0.04).
